# Plasma protein levels of young healthy pigs as indicators of disease resilience

**DOI:** 10.1093/jas/skad014

**Published:** 2023-01-13

**Authors:** Yulu Chen, Steven Lonergan, Kyu-Sang Lim, Jian Cheng, Austin M Putz, Michael K Dyck, PigGen Canada, Frederic Fortin, John C S Harding, Graham S Plastow, Jack C M Dekkers

**Affiliations:** Department of Animal Science, Iowa State University, Ames, IA, USA; Department of Animal Science, Iowa State University, Ames, IA, USA; Department of Animal Science, Iowa State University, Ames, IA, USA; Department of Animal Resources Science, Kongju National University, Yesan, Republic of Korea; Department of Animal Science, Iowa State University, Ames, IA, USA; Department of Animal Science, Iowa State University, Ames, IA, USA; Hendrix Genetics, Swine Business Unit, Boxmeer, The Netherlands; Department of Agriculture, Food and Nutritional Science, University of Alberta, Edmonton, AB, Canada; PigGen Canada Research Consortium, Guelph, Ontario, Canada; Centre de Développement du Porc du Québec Inc., Québec City, Canada; Department of Large Animal Clinical Science, University of Saskatchewan, Saskatoon, SK, Canada; Department of Agriculture, Food and Nutritional Science, University of Alberta, Edmonton, AB, Canada; Department of Animal Science, Iowa State University, Ames, IA, USA

**Keywords:** disease resilience, indicator traits, pigs, plasma proteins

## Abstract

Selection for disease resilience, which refers to the ability of an animal to maintain performance when exposed to disease, can reduce the impact of infectious diseases. However, direct selection for disease resilience is challenging because nucleus herds must maintain a high health status. A possible solution is indirect selection of indicators of disease resilience. To search for such indicators, we conducted phenotypic and genetic quantitative analyses of the abundances of 377 proteins in plasma samples from 912 young and visually healthy pigs and their relationships with performance and subsequent disease resilience after natural exposure to a polymicrobial disease challenge. Abundances of 100 proteins were significantly heritable (false discovery rate (FDR) <0.10). The abundance of some proteins was or tended to be genetically correlated (*r*_g_) with disease resilience, including complement system proteins (*r*_g_ = −0.24, FDR = 0.001) and IgG heavy chain proteins (*r*_g_ = −0.68, FDR = 0.22). Gene set enrichment analyses (FDR < 0.2) based on phenotypic and genetic associations of protein abundances with subsequent disease resilience revealed many pathways related to the immune system that were unfavorably associated with subsequent disease resilience, especially the innate immune system. It was not possible to determine whether the observed levels of these proteins reflected baseline levels in these young and visually healthy pigs or were the result of a response to environmental disturbances that the pigs were exposed to before sample collection. Nevertheless, results show that, under these conditions, the abundance of proteins in some immune-related pathways can be used as phenotypic and genetic predictors of disease resilience and have the potential for use in pig breeding and management.

## Teaser

Plasma protein levels of young healthy pigs are promising as biomarkers to select for disease resilience.

## Introduction

Infectious diseases directly take their toll on the swine industry by increasing mortality, reducing productivity and animal welfare, and are among the main obstacles and sources of losses in pork production (Morgan and Prakash, 2006). For example, in 2013, Holtkamp et al. (2013) estimated porcine reproductive and respiratory syndrome to cost the U.S. swine industry around $664 million annually and this disease continues to be a major problem in the swine industry in North America and globally.

For the past three decades, goals for pig breeding have focused primarily on lean meat growth, feed efficiency, carcass quality, and reproduction. In recent years, however, the goal of pig breeding has expanded to include health-related traits such as disease resilience (Mellencamp et al., 2008), which refers to the ability of an animal to maintain relatively undiminished performance when exposed to diseases (Albers et al., 1987; Bisset and Morris, 1996; Nguyen-Ba et al., 2020). Incorporating health traits into breeding programs can reduce economic losses due to disease, increase production efficiency, reduce the use of antibiotics, and improve animal welfare. Disease pressures on commercial pig farms are complex, so selective breeding of pigs that are more resilient across common diseases can be a practical way to improve productivity (Putz et al., 2019).

In most cases, direct selection for disease resilience in breeding programs is not feasible because nucleus breeding stock is by necessity kept in high-health bio-secure environments. Quantifying disease resilience to polymicrobial infectious agents in commercial farms is also challenging because it is a manifestation of many biological processes, is simultaneously affected by multiple factors, and collecting individual animal data in commercial farms is complex. A promising alternative is an indirect selection based on a suitable indicator trait of disease resilience that can be measured on healthy animals, i.e. in the nucleus, preferably at a young age. For an indicator trait for disease resilience to be effective, it needs to be measurable in a nucleus environment, heritable, and genetically correlated with disease resilience.

Proteins are downstream products of the genome that drive many cellular processes (Lesk, 2001). The blood proteome is a complex composite of proteins, including classical blood proteins and proteins secreted or leaked from tissues, including hormones, cytokines, adipokines, chemokines, and growth factors that coordinate biological processes associated with health or diseases (Lin et al., 2008). The blood proteome, thereby, provides a window into the current state of the body, including health (Anderson et al., 2004).

By applying machine learning to large-scale and deep plasma proteome data, Williams et al. (2019) demonstrated that plasma proteome patterns can be a comprehensive predictor of human life span and health status, including future age-related disease risks. In addition, colocalization of DNA variants that are associated with diseases with the genomic location of genes for intermediate phenotypes such as blood protein levels can be used to identify drug targets and disease-translational biomarkers (Kastenmuller et al., 2015; Suhre et al., 2021).

Previous studies have shown that the abundance of some proteins in blood is heritable or regulated by genetic factors, including in humans (Johansson et al., 2013; Liu et al., 2015), mice (Holdt et al., 2013), dairy cattle (Cecchinato et al., 2018), and pigs (Clapperton et al., 2009; Reyer et al., 2019; Ballester et al., 2020). In pigs, some studies have shown that the blood proteome changes with disease status (te Pas et al., 2013; Muk et al., 2019) and that the levels of some plasma proteins, such as alpha-acid glycoproteins, are genetically negatively correlated with average daily gain (from −0.72 to −0.53) (Clapperton et al., 2009).

Here, we integrated the population-level plasma proteome of young, visually healthy pigs with whole-genome single-nucleotide polymorphism (SNP) genotype data and extensive phenotypes measured before and after their exposure to a polymicrobial natural disease challenge. The overall objective was to explore whether the plasma proteome of young, healthy piglets can be early indicators for disease resilience by (1) investigating the genetic basis of the plasma proteome of young, healthy pigs, and (2) identifying phenotypic and genetic associations of the plasma proteome of young, healthy pigs with their subsequent disease resilience phenotypes, as well as the biological basis behind these associations.

## Materials and Methods

### Ethical statement

The project protocol was approved by the Animal Protection Committee of the Centre de Recherche en Sciences Animales de Deschambault (15PO283) and the Animal Care and Use Committee of the University of Alberta (AUP00002227) and carried out following Canadian Council on Animal Care guidelines (CCAC; https://ccac.ca/en/guidelines-and-policies/fundamental-principles.html). The Quebec Provincial Centre for Population Development and pastoralists and project veterinarians provided comprehensive oversight of animal care. Pigs in this project were humanely euthanized when humane intervention points were exceeded or response to treatment was inadequate. Following CCAC guidelines, electrocution was used to euthanize pigs during the nursery period, while a captive cranial bolt was used during the finisher period. According to standard approved industry protocols, pigs that reached slaughter weight were stunned by electrocution at a commercial slaughter facility, followed by exsanguination.

### Natural disease challenge model

This study used data from the polymicrobial natural disease challenge model (NDCM) described by Putz et al. (2019), which was established in 2015 and continued until 2021 at the CDPQ in Québec, Canada, to study the genetic control of disease resilience in grow-finish pigs. The NCDM was designed to simulate the disease pressure in a commercial farm with poor health and was established by bringing naturally infected pigs into a nursery and finisher barn and maintained by continuous flow, with older batches of pigs exposing incoming batches to diseases by nose-to-nose contact. Every 3 weeks, a batch of 60 or 75 weaned, Large White by Landrace crossbred barrows was provided from a bio-secure multiplier farm in Canada from one of the seven members of PigGen Canada (http://piggencanada.org/), in rotation. One rotation of seven batches (one batch per company) was referred to as a cycle, for a total of seven cycles. Further details are in Putz et al. (2019).

Performance (prior to challenge) and resilience (after challenge) phenotypes were collected on all pigs over three growth phases, as described by Putz et al. (2019): (1) quarantine nursery phase (on average 19 days, starting at ~21 days of age); (2) challenge nursery phase (on average 28 days, starting at ~40 days of age); and (3) finisher phase (on average 100 days, starting at ~70 days of age). Detailed performance and resilience phenotypes were available on 3,205 pigs from cycles 1 to 7, as described by Putz et al. (2019) and Cheng et al. (2020) and included: average daily gain in the quarantine nursery (qNurADG), in the challenge nursery (cNurADG), and in the finisher (cFinADG); the number of individual parenteral antibiotic treatments provided in the challenge nursery, adjusted to 27 days (cNurTRT), in the finisher, adjusted to 100 days (cFinTRT), and from birth, adjusted to a standard 180 days of age at slaughter (AllTRT); mortality (0 for pigs that survived; 1 for pigs that died) in the challenge nursery (cNurMOR), in the finisher (cFinMOR), and across the challenge nursery and finisher phases (AllMOR); subjective health scores (HS) assigned by trained personnel on a 1–5 scale based on clinical signs (1 = severe clinical signs to 5 = perfect health, see Cheng et al. (2020)) at 5 and 19 days after entry into the quarantine nursery (qNurHS1 and qNurHS2), at 3 weeks after entry into the challenge nursery (cNurHS), and at 6 weeks after entry into the finisher (cFinHS); average daily feed intake (ADFI), feed conversion ratio (FCR), and residual feed intake (RFI) in the finisher; and carcass weight (CWT), carcass back fat (CBF), carcass loin depth (CLD), dressing percentage (DRS), and lean yield (LYD) at slaughter. Incomplete phenotypes for cFinADG, cFinTRT, AllTRT, ADFI, and FCR for pigs that died in the finisher were imputed and expanded as described by Cheng et al. (2020), to put them on the same scale as those of pigs that survived to slaughter. This resulted in two data sets for these traits: survivor data, which only included phenotypes on pigs that survived to slaughter, and expanded data, which also included imputed data on selected pigs that died in the finisher (see Cheng et al. (2020) for details). Because of the limited number of animals receiving low health scores (see Cheng et al. (2020) for details) and because the scale of scores may not be linear, pigs with an HS less or equal to 4 were assigned a score of 4. Detailed statistics and estimates of genetic parameters of these performance and resilience phenotypes are in Cheng et al. (2020).

### Genotyping and quality control

All animals were genotyped for 658,692 SNPs using a 650 k Affymetrix Axiom Porcine Genotyping Array by Delta Genomics (Edmonton AB, Canada). Raw data were processed separately for each cycle by Delta Genomics, using default settings of the Axiom Analysis Suite (quality control thresholds: call rate for marker > 0.10; call rate for individual > 0.10; minor allele frequency > 0.05), as described by Putz et al. (2019). After quality control, 435,172 SNPs on 3,205 pigs remained for further analysis.

### Protein abundance measurement

Whole blood samples were collected ~5 days after entering the quarantine nursery (~26 days of age) into K2 ethylenediaminetetraacetic acid (EDTA) tubes (BD Vacutainer Blood Collection Tubes, United States). Multiple studies have shown that physiological indicators at this time may also be affected by weaning and transportation (Zhu et al., 2012; Buchet et al., 2017; Montagne et al., 2022), so we regard pigs at this time as visually healthy weaned pigs and we use “healthy” to refer to “visually healthy weaned” in the remainder. After centrifugation (2,000 g at 4 °C for 10 min) the plasma layer was aliquoted by transfer pipette into Thermo Scientific Nunc barcoded tubes. Immediately after processing, samples were frozen at −80 °C until subsequent analysis. The available samples were processed in two groups (groups 1 and 2), in December 2018 and November 2019. Samples in group 1 came from cycles 4 and 5, while samples in group 2 came from cycles 4 to 7. The protein content in each blood sample was determined (Bradford, 1979) using pre-mixed reagents (Bio-Rad Laboratories, Hercules, CA) and adjusted to 10 µg/µL. Protein abundances, quality, and profiles were evaluated using 15% SDS–PAGE gels and Colloidal Coomassie blue staining (1.7% ammonium sulfate, 30% methanol, 3% phosphoric acid, and 0.1% Coomassie G-250) (Cruzen et al., 2015). Samples were stored at −80 °C until labeling and analysis.

The Thermo Scientific TMT Mass Tag Labeling Kits and Reagents protocol (11-plex) was used to identify and quantify the abundance of individual proteins in each plasma sample (Thompson et al., 2003). For each sample, 25 μg was diluted with 50 mM Tris (pH 8) to a concentration of 0.5 μg/μL. Then, 5 μL of 0.1 M DTT was added to reach a final concentration of 5 mM, and samples were mixed and incubated at 37 °C for 30 min. The alkylation process was conducted by adding 1.5 μL of 1M iodoacetamide to a final concentration of 15 mM, after which samples were mixed and incubated in the dark at room temperature for 30 min. Then, 400 μL of 50 mM Tris–HCl (pH 8) was added to dilute samples, which reduced the concentration of urea for optimal trypsin activity. Trypsin was added to each sample at a 1 μg trypsin:50 μg sample ratio and incubated overnight at 37 ºC. The digestion process was stopped by adding formic acid (5 μL) to a final concentration of 1%. Samples were then centrifuged at 14,000–16,000 × *g* for 10 min using a benchtop microcentrifuge to remove particulate material, after which the samples were desalted using the Microspin column (SEM SS18V) and dried down using the SpeedVac. The tryptic peptide samples were reconstituted in 100 μL with 50 mM triethyl ammonium bicarbonate), mixed with 0.2 mg (10 μL) of the corresponding labeling reagent, and incubated for 1 h. The samples were then quenched with 8 μL of 5% hydroxylamine (50 μL hydroxylamine in 450 μL 100 mM TEAB) and incubated for 15 min. The TMT 11-plex system can conduct quantitation using high-resolution MS for 11 samples simultaneously, which we refer to as a run. Within a run, each of the 11 samples had a unique labeling tag (126, 127N, 127C, 128N, 128C, 129N, 129C, 130N, 130C, 131N, and 131C). For each of the two groups of samples a reference sample was created by pooling 10 µg from each sample in the group, which was labelled with tag 131C in each run. For each run, the 11 labelled peptide samples were mixed, then eluted in 55 μL 5% acetonitrile and 0.1% formic acid, and dried by vacuum.

Peptides were separated by liquid chromatography (Thermo Scientific EASY nLC-1200 coupled to a Thermo Scientific Nanospray FlexIon source) through a pulled glass emitter 75 µm × 20 cm (Agilent capillary, part #16-2644-5). The tip of the emitter was packed with C18 packing material (Agilent Zorbax Chromatography Packing, SB-C18, 5 µm, part #8220966-922), while the remainder of the column was packed with UChrom C18 3 micron material from nanoLCMS Solutions (part #80002). In detail, buffer A was 0.1% formic acid in the water and buffer B was 0.1% formic acid in 80% acetonitrile/water. The gradient was comprised of an increase from 0% to 35% B in 210 min, followed by an increase to 70% B in 20 min, then an increase to 100% B in 5 min. The flow rate for the equilibration and separation was 300 nL/min. ESI voltage was at 2.65 kV in positive polarity mode. Subsequently, the peptides were fragmented for analysis by MS/MS using a Thermo Scientific Q Exactive Hybrid Quadrupole-Orbitrap Mass Spectrometer with an HCD fragmentation cell (Waltham, MA) using a Full MS/DD-MS2 (TOPN) method. Full MS scans were run with a scan range of 400–2000 m/z at a resolution of 70,000, with an AGC target of 1e^6^ and a maximum IT of 80 ms. The top 20 MS2 scans were run at a resolution of 35,000 with an AGC target of 1e^5^ and a maximum IT of 50 ms. The isolation window was set to 1.2 m/z and the fixed first mass was set to 110.0 m/z. The NCE setting was at 32.

The acquired raw data were analyzed using the Proteome Discoverer software (version 2.4; Thermo Fisher Scientific, San Jose, CA, USA), separately for the groups 1 and 2 samples. Each raw file was searched against a Sus scrofa FASTA file database (UniProtKB database) on Sequest HT and Mascot search engines. Both searches were performed with a static modification of carbamidomethyl (Cys) and TMT label (Lys and N-termini), along with dynamic modifications of oxidation (Met) and deamidation (Asn, Gln). For both search engines, peptide spectral matches were validated at an FDR of 1% under the percolator of the Proteome Discoverer software. The precursor mass tolerance was set at 10 ppm and the fragment mass tolerance at 0.02 Da for both search engines. The identified proteins were required to have at least one peptide sequence detected.

The plasma samples were processed in two groups, resulting in some differences in the proteins detected for the two groups. Samples in groups 1 and 2 were, respectively, evaluated in 41 and 51 TMT 11-plex runs. Proteins detected also differed between runs but were confounded within a run, i.e. if one sample in one run had a missing value for a protein, then all samples in that run had a missing value for this protein. As a result, the percent of missingness increased significantly when all samples were combined, which was also observed by Bramer et al. (2021) and Brenes et al. (2019). Missingness was, however, not associated with the level of abundance of the protein and could be considered random (see Supplementary Figure S1). The processed datasets from the two groups were then merged and proteins that were identified in more than 20 runs were used for statistical analysis.

### Statistical analyses

To improve normality, the protein abundance data were transformed using the logarithm function with base 2. In Supplementary Figure S1, there was no relationship between the frequency of missing values for a protein vs. the average of the log2 protein abundance for the samples in runs for which that protein was not missing. Hence, the missing data were assumed to be missing at random in subsequent analyses.

For each filtered protein, the following mixed linear model (1) was used to estimate the residual adjusted for systematic effects for each protein abundance observation:


$$\begin{array}{ll} y_{ijklm}= & Batch_{i}+\;Tag_{j}+\;EntryAge_{ijklm}+\;ref\;{\left(group\;1\right)}_{ijklm}+\\ & \;ref\;{\left(group\;2\right)}_{ijklm}+Plex_{l}+e_{ijklm} \end{array}$$ (1)

where $y_{ijklm}$ is the log_2_ transformed protein abundance; $Batch_{i}$ and $Tag_{j}$ are the fixed effects of batch (i=1, 2, …, 50) and TMT tag (*j* = 1, 2, …, 10), respectively; $EntryAge_{ijklm}$ is the covariate of age at entry into the quarantine nursery; $ref{\left(group\;1\right)}_{ijklm}$ and $ref\;{\left(group\;2\right)}_{ijklm}$ are the log2 transformed abundances of the reference sample for run *m* for groups 1 and 2, respectively, which were fitted as covariates ($ref{\left(group\;1\right)}_{ijklm}\;$ was set equal to 0 for runs for group 2, and vice versa); $Plex_{l}$ is the random effect of run, assumed to be distributed $N(0,\;I\sigma_{pl}^{2})$, where $\sigma_{pl}^{2}$ is the run variance; and $e_{ijklm}$ is a random residual, which was assumed to be distributed $N(0,\;I\sigma_{e}^{2})$, where $\sigma_{e}^{2}$ is the residual variance. For each protein, residuals that were outliers based on the 1.5 × inter-quartile range rule were removed (around 3% of observations).

#### Phenotypic associations with performance and resilience

Associations of the abundance of each protein with each of the recorded performance and resilience traits were analyzed using the following mixed linear model, separately for each protein:


$$\begin{array}{ll} y_{ijkm}= & Batch_{i}+\;EntryAge_{ijkm}+\;Protein_{ijkm}\\ & +Pen_{j}+litter_{ijk}+\;e_{ijkm} \end{array}$$ (2)

where $y_{ijkm}$ is the phenotype for a performance or resilience trait for a pig with proteome data; $Protein_{ijkm}$ is the protein residual from model (1), which was fitted as a covariate, one protein at a time; $litter_{ijk}$ is the random effect of common litter environmental effects, assumed to be distributed $N(0,\;I\sigma_{l}^{2})$, where $\sigma_{l}^{2}$ is the litter variance, and all other effects are as described for model (1), except that $Pen_{j}$ refers to the pen corresponding to the time period of $y_{ijk}$, as described in Cheng et al. (2020). For carcass traits, slaughter date was added as a fixed effect, as well as the covariates of age and weight at slaughter. For categorical traits (i.e. health scores and mortality), a reverse mixed linear model was used to analyze the association between protein abundance and the trait phenotype because logistic regression analyses of these traits failed to converge in some cases. In the reverse mixed linear model analyses, the protein residual from model (1) was used as the response variable and the binary phenotype (0/1) was fitted as a covariate in a mixed linear model (2). The resulting estimates of the regression coefficient of the binary trait on protein abundance were then converted to regression coefficients of protein abundance on the binary trait by multiplying the estimate by the ratio of the variances of the residuals of the binary trait and of protein abundance. The Benjamini and Hochberg (1995) method was used to estimate the FDR of *P*-values across all recorded phenotypes and tests. To ensure sufficient proteins were available for downstream analyses, estimates with FDR less than 0.35 considered statistically significant. These analyses were implemented in the R packages lme4 (Bates et al., 2014) and car (Fox and Weisberg, 2019).

#### Heritability of protein abundance

A model similar to model (1) was used to estimate the variance components and genetic parameters for abundance of each protein but with the addition of random litter effects ($litter_{ikm}$, assumed distributed $N\;(0,\;I\sigma_{l}^{2})$, where $\sigma_{l}^{2}$ is the litter environmental variance) and random animal additive genetic effects ($a_{ijkmu}$), which were assumed to be distributed $N(0,\;G\sigma_{a}^{2})$, where G is the genomic relationship matrix and $\sigma_{a}^{2}$ is the additive genetic variance. Matrix ***G*** was created based on the SNP genotype data using the PreGSf90 software of BLUPF90 (Misztal et al., 2002). This matrix was computed separately for pigs from each company and then combined, with the relationship between pigs from different companies set to 0 to focus on pooled within-company variances, as described by Cheng et al. (2020). Variance components were estimated using restricted maximum likelihood using the ASReml 4.0 software (Gilmour et al., 2014). Estimates of heritability and of the proportion of variance due to the litter effects were calculated as a proportion of phenotype variance, which was the sum of estimates of $\sigma_{e}^{2}$, $\sigma_{l}^{2}$, and $\sigma_{a}^{2}$. A likelihood ratio test based on the difference in likelihood between models with and without additive genetic effects was used to determine the significance from zero estimates of heritability and of the proportion of variance due to litter effects. The *P*-value for this test was obtained by comparison to a Chi-square distribution with one degree of freedom, with the resulting *P*-value divided by 2 because the maximum likelihood estimates were restricted to be positive (Visscher, 2006). Variance ratios with FDR (Benjamini and Hochberg, 1995) less than 0.10 across proteins were considered significant.

#### Genetic correlations of protein abundance with performance and resilience traits

Genetic correlations of the abundance of proteins with each performance and resilience trait were estimated using bivariate models in ASReml 4.0. Genetic correlations were only estimated for proteins whose abundance had an heritability estimate larger than 0.05 because bivariate analyses often fail to converge or lead to estimates of genetic correlations with extremely large SE if one of the traits has a low heritability. Genetic correlations were estimated using the proteome data on the 912 animals and performance and resilience data from pigs in all 50 batches (3205 animals), with the full genomic relationship matrix, created as described by Cheng et al. (2020). The model used for proteome abundance was the same as that used to estimate heritability. For the performance and resilience traits, model (2) was used but with protein residual removed as a covariate and an animal additive genetic effect added as a random effect, as described in Cheng et al. (2020). To determine significance of genetic correlation estimates from zero, a likelihood ratio test (Visscher, 2006) for models with and without the genetic correlation set to 0 was used, with *P*-values obtained by comparison to a Chi-square distribution with one degree of freedom. The Benjamini and Hochberg (1995) method was used to estimate the FDR of *P*-values across all phenotypic traits. To ensure sufficient proteins were available for downstream analyses, estimates of genetic correlations with FDR less than 0.35 were considered to be statistically significant.

#### Gene set enrichment analyses

The GSEAPreranked tool of the GSEA_4.1.0 software (Subramanian et al., 2005) was used to perform gene set enrichment analysis of estimates of phenotypic associations of protein abundance with performance and resilience phenotypes from model (1). For these analyses, the Gene Ontology (GO) biological process library and the REACTOME pathway library based on UniProt protein ID were used as protein annotation databases in separate analyses. The GO biological process library was built based on the current pig GOA (https://www.ebi.ac.uk/GOA/pig_release, released on 14 August 2020), with 10,627 GO biological processes. The REACTOME pathway library was downloaded from https://reactome.org/download-data (accessed on August 2020), with 1,562 pathways for swine. The format of the two libraries was adjusted to the GSEA GMT format. The GSEA analyses were conducted separately for each resilience trait, with the proteins ranked based on regression coefficient estimates of the phenotype on protein abundance residuals from model (1), and corresponding estimates from the reverse linear mixed models for categorical traits. For regression coefficients to be comparable across traits and proteins, they were scaled by multiplying the estimate by the ratio of the SD of protein abundance residuals and the SD of the resilience trait phenotype, such that they were expressed in units of (SD) of the resilience trait per SD of protein expression residual. The GSEA software was run using the UniProt protein ID’s of the libraries as “gene sets,” with the following settings: number of permutations = 1,000; no collapse; enrichment statistic = weighted; max size for excluding larger sets = 500; min size for exclude smaller sets = 1. The FDR and normalized enrichment scores for each set and trait were obtained from the GSEA output.

A similar procedure was used for GSEA of estimates of genetic correlations between proteins and performance and resilience traits, except that proteins were ranked by the signed −log10 of the *P*-value of the likelihood ratio test for the estimate of the genetic correlation, with the sign reversed if the genetic correlation estimate was negative to provide a direction to the enrichment associations.

REACTOME pathway or GO terms with FDR below a chosen threshold for at least one resilience trait (chosen to ensure sufficient terms were available for subsequent analyses) were clustered using the ward.D method (Ward Jr, 1963), separately for the phenotypic and genetic enrichment analyses. This clustering was based on the signed −log10(FDR) of enrichment of these terms or pathways for each trait, where the sign was based on whether an increase in abundance of core proteins in the REACTOME pathway or GO terms were associated with a favorable (+) or an unfavorable (−) change in the trait phenotype based on the estimate of the corresponding regression coefficient or genetic correlation. For this purpose, the signs of the estimates for TRT, MORT, FCR, RFI, and CBF were reversed because lower values are favorable for these traits. The R package ComplexHeatmap (Gu et al., 2016) was used to visualize the GSEA results.

## Results

**Figure 1.**
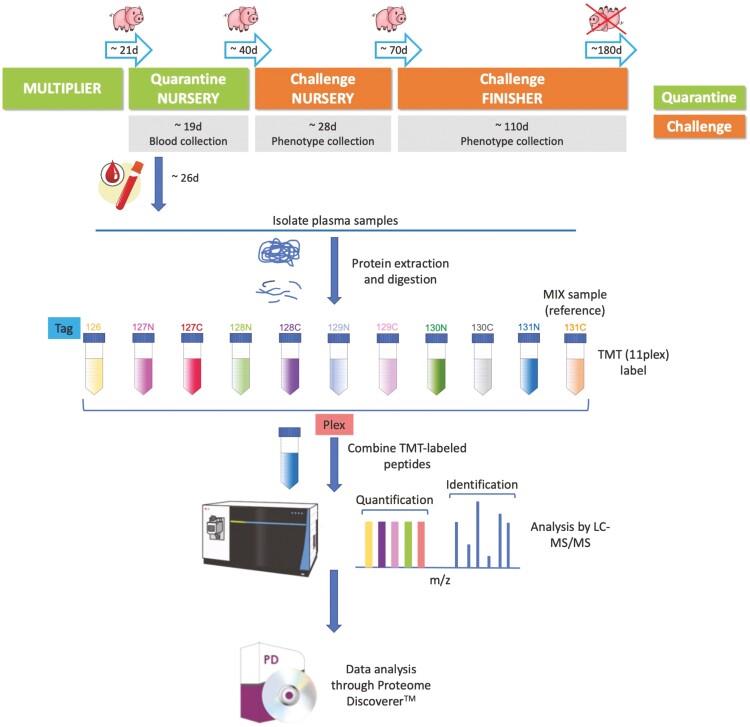
Overview of the quantification of the plasma proteome of young healthy pigs using TMT. Experimental design and TMT workflow. Plasma samples were collected in the quarantine nursery on 912 pigs. The plasma proteome was measured by TMT for two groups of samples. For each group, a pool of all samples in the group was labeled with tag 131C and included in each 11-plex, along with 10 samples with unique tags, which were pooled and fractionated by high-pH liquid chromatography. All fractions were analyzed on the Thermo Scientific Q Exactive Hybrid Quadrupole-Orbitrap Mass Spectrometer. Acquired raw data were analyzed using the Proteome DiscovererTM software.

This study used data and samples from the polymicrobial natural disease challenge model (NDCM) for grow-finish pigs described by Putz et al. (2019). Performance and resilience phenotypes collected are summarized in Table 1. Proteome data were obtained on plasma samples collected in a quarantine nursery on 912 pigs, prior to their entry into the disease challenge, as illustrated in Figure 1. The plasma samples were processed in two groups, with details in Table 2. After filtering, 377 proteins that were identified in more than 20 runs were used for further analyses. Distributions of raw protein abundances, of log2 transformed protein abundances, and of residuals of log2 protein abundances adjusted for nuisance effects (see later) are shown in Supplemental Figure S2 for randomly selected proteins. Distributions of the residuals of log2 abundances were close to normal.

**Table 1.** Summary of the phenotypic data from the natural disease challenge model

Phase and trait	Abbreviation	Number of records
Quarantine nursery	*qNur*	
Average daily gain, kg/day	qNurADG	3,131
Health score 1	qHScore1	3,110
Health score 2	qHScore2	2,960
Challenge nursery	*cNur*	
Average daily gain, kg/day	cNurADG	3,112
Health score	cHScore	3,013
Mortality, 0/1 = died	NurMOR	3,120
Number of treatments	NurTRT	3,098
Finisher	*Fin*	
Average daily gain, kg/day	FinADG	2,295 (2,338^1^)
Average daily feed intake, kg/day	ADFI	2,292 (2,332)
Average daily feed duration, min/day	ADFD	2,292 (2,374)
Feed conversion ratio	FCR	2,291 (2,333)
Health score	FinHScore	2,544
Mortality, 0/1 = died	FinMOR	2,774
Number of treatments	FinTRT	2,295 (2,548)
*** *** Challenge nursery + finisher	*All*	
Mortality, 0/1 = died	AllMOR	3,139
Number of treatments	AllTRT	2,295 (2,662)
Carcass traits		
Carcass weight, kg	CWT	2,124
Carcass backfat, mm	CBF	2,005
Carcass loin depth, mm	CLD	2,007
Lean yield, kg	LYLD	2,002
Dressing percentage, %	DRS	2,120

^1^ Number of pigs in the expanded data, following Cheng et al. (2020). All other counts refer to pigs that survived to the end of the respective phase (except counts for mortality).

**Table 2.** Summary of the proteome data

	P727 dataset (group 1)	P834 dataset (group 2)
Measurement time	December 2018	November 2019
No. of runs	41	51
No. of samples	451	561
No. of proteins detected	481	791
Cycle^1^	4, 5	4, 5, 6, 7

^1^The cycles of the natural disease challenge model (Cheng et al., 2020) that the samples were from.

### Phenotypic associations of protein abundance with performance and resilience phenotypes

**Figure 2.**
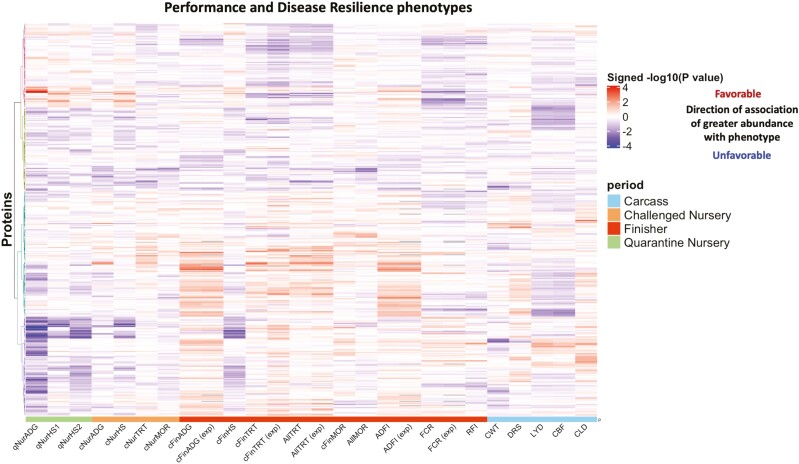
Heat map of the signed −log10(*P*-value) for phenotypic associations of protein abundance in blood of young healthy pigs with concurrent and subsequent performance and disease resilience phenotypes from the survivor and expanded (exp) data sets. For trait abbreviations, see Table 1. The colors above the traits indicate the time period that the trait measured, where green represents quarantine nursery, orange the challenge nursery, red the finisher, and blue slaughter. Red/blue of the heat map value indicates that an increase in abundance of that protein is favorable/unfavorably associated with the performance or disease resilience phenotype. Colors on the dendrogram identified different clusters based on ward.D clustering.

Associations of the abundance of individual proteins in plasma of young, healthy pigs with their concurrent and subsequent performance and resilience phenotypes were analyzed and are presented in Figure 2 as a heatmap of the signed −log10 of the *P*-value of the phenotypic association. For a given protein and trait, the signed −log10 (*P*-value) was highly correlated with the corresponding scaled regression coefficients of protein abundance on trait phenotype, as shown in Supplementary Figure S3. Table 3 shows proteins that were significantly associated with at least one performance or resilience phenotype at an FDR less than 0.35 across all proteins and analyzed phenotypes.

**Table 3.** Proteins with abundance levels in blood of young healthy pigs that were phenotypically significantly (FDR < 0.35) associated with performance and resilience phenotypes

Trait	UniProt ID	Protein name	Gene name	Standardized coefficient estimate^1^	*P*-value^2^	FDR^3^	Heritability
qNurADG^4^	A0A4X1VPB7	Inter-alpha-trypsin inhibitor heavy chain H4	*ITIH4*	−0.02	1.18E−06	0.01	0.02
qNurADG	F1SFI5	Histidine rich glycoprotein	*HRG*	0.01	3.28E−06	0.02	0.14
qNurADG	A0A4X1TQP5	Glutathione peroxidase		−0.01	4.07E−05	0.13	0.15
qNurADG	A0A4X1U6L2	Uncharacterized protein		−0.02	5.75E−05	0.14	0.33
qNurADG	A0A4X1VBD2	C4a anaphylatoxin (Complement C4 gamma chain)	*C4A*	−0.02	7.76E−05	0.15	0.30
qNurADG	F1SFI6	Fetuin B	*FETUB*	0.01	1.35E−04	0.22	0.00
qNurHS1^5^	A0A4X1U6T3	Uncharacterized protein		−0.15	2.83E−04	0.31	0.51
cNurHS^6^	A0A4X1U6T3	Uncharacterized protein		−0.16	2.62E−04	0.31	0.51
cFinHS^7^	F1STC5	Ig-like domain-containing protein		−0.34	2.50E−04	0.31	0.09
cFinADG (exp)^8^	A0A4X1SMT6	Vitamin K-dependent protein C	*PROC*	0.02	3.57E−04	0.35	0.21

^1^Standardized the coefficient estimates by multiplying them by the SD of the protein residuals, such that the coefficient estimates refer to the increase in the trait per 1 SD increase in log2(protein abundance).

^2^ *P*-value of the association of protein abundance with the phenotypic trait.

^3^FDR of the association of protein abundance with the phenotypic trait across all evaluated phenotypic traits.

^4^Average daily gain during the quarantine nursery period.

^5^Health score 1 measured during the quarantine nursery period.

^6^Health score measured during the challenge nursery period.

^7^Health score measured during the finisher period.

^8^Average daily gain during the finisher period for the expanded data; all other results are for the survivor data.

Abundance of several proteins had relatively strong associations with some traits, especially with traits that were recorded during the phase when the samples for proteome analysis were collected, e.g. average daily gain (ADG; see Table 1 for abbreviations) in the quarantine nursery and subjective health scores (HS) taken at two time points in the quarantine nursery (qNurHS1 and qNurHS2). These three phenotypes had relatively similar association patterns with protein abundances (Figure 2). Health score recorded in the challenge nursery had similar association patterns with protein abundances as the first health score in the quarantine nursery (Figure 2). The number of health treatments a pig received during the different phases, i.e. in the challenge nursery (cNurTRT), the finisher (cFinTRT), and across the challenge nursery and finisher (AllTRT), also showed similar association patterns to each other (Figure 2), recognizing that AllTRT has a part-whole relationship with cNurTRT and cFinTRT. Associations with mortality (MOR) during the different periods were rather weak and inconsistent. For traits in the finisher, association patterns for analyses based on data of pigs that survived to slaughter (survivor data) and data that included selected pigs that died (expanded data, see Table 1) were very similar.

### Heritability of protein abundance

**Figure 3.**
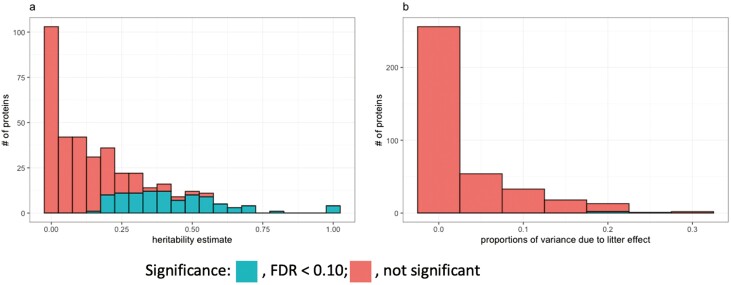
Histograms of (a) heritability estimates and (b) proportions of variance due to litter effects for the abundance of each protein in the blood of young healthy pigs. The green color indicates estimates with FDR less than 0.10.

Figure 3 shows a histogram of estimates of heritability and of the proportion of variance due to litter effects for the abundance of each protein in the blood. Heritability estimates were significant (FDR < 0.10) for 100 of the 377 proteins, ranging from 0.17 to close to 1. The top four heritable proteins, with estimates close to 1, were A0A480TLF3, A0A480P4D2, A0A286ZKB4, and A0A4X1T2W4. Litter effects were significant (FDR < 0.10) for only two proteins.

### Genetic correlations of protein abundance with performance and resilience traits

**Figure 4.**
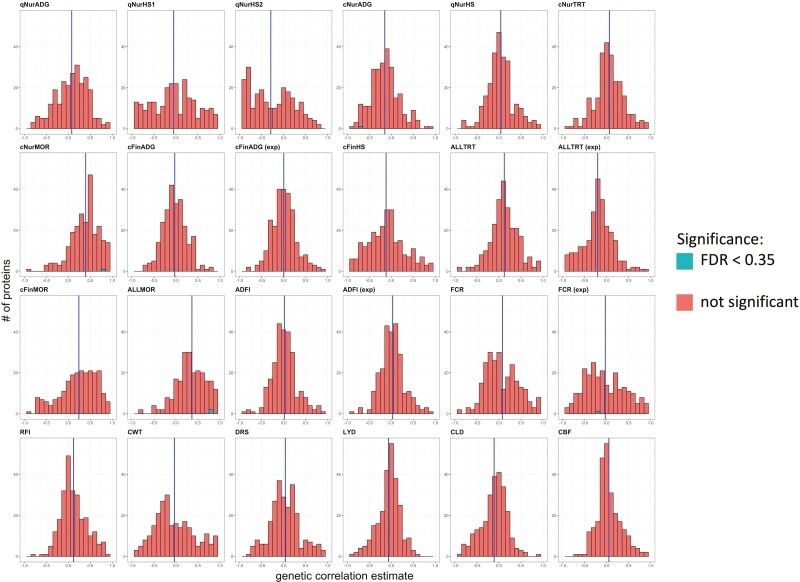
Histograms of estimates of genetic correlations of protein abundance in blood of young healthy pigs with performance and disease resilience phenotypes. For trait abbreviations, see Table 1. The green color indicates estimates with FDR less than 0.35. The blue vertical line is the mean of the genetic correlation estimates for that trait.

Histograms of estimates of genetic correlations of the abundance of 193 proteins that had heritability estimates greater than 0.05 with performance and resilience traits are shown in Figure 4. The abundance of five proteins had significant non-zero genetic correlations with is trait at the liberally chosen threshold of FDR < 0.35 (Table 4). No proteins were significant for more than one trait. For all but four traits (qNurHS2, cNurMOR, cFinMOR, and AllMOR), the mean of the genetic correlation estimates across proteins markedly deviated from zero. For example, the mean of genetic correlation estimates across proteins was positive with mortality during the different phases and negative with qNurHS2. This might be because these binary traits were treated as continuous variables in these analyses.

**Table 4.** Proteins with abundance levels in blood of young healthy pigs that were genetically significantly (FDR < 0.35) correlated with performance and resilience phenotypes

Trait	UniProt ID	Protein name	Gene name	Genetic correlation^1^	*P*-value^2^	FDR^3^	Heritability
cNurADG^4^	L8B0V2	IgG heavy chain	*IGHG*	−0.68	2.20E−04	0.22	0.39
cNurMOR^5^	L8AXM5	IgG heavy chain	*IGHG*	0.85	2.12E−04	0.19	0.58
ALLMOR^6^	A0A286ZWN6	Uncharacterized protein		0.82	5.21E−04	0.33	0.43
ALLMOR	F1RX36	Fibrinogen alpha chain	*FGA*	0.81	5.32E−04	0.33	0.27
FCR (exp)^7^	A0A4X1T2W4	Complement component C9	*C9*	−0.24	6.49E−07	0.001	1.00

^1^Estimate of the genetic correlation of protein abundance with the phenotypic trait, with the sign indicating whether an increase in abundance is associated with a favorable (+) or unfavorable (−) change in the phenotype.

^2^ *P*-value for the likelihood ratio test of the estimate of the genetic correlation.

^3^FDR for the likelihood ratio test of the estimate of the genetic correlation across all evaluated phenotypic traits.

^4^Average daily gain during the challenge nursery period.

^5^Mortality (0/1 = survived during/died during the challenge nursery period).

^6^Mortality (0/1 = survived to/died prior to slaughter).

^7^Feed conversion ratio for the expanded data; all other results are for the survivor data.

**Figure 5.**
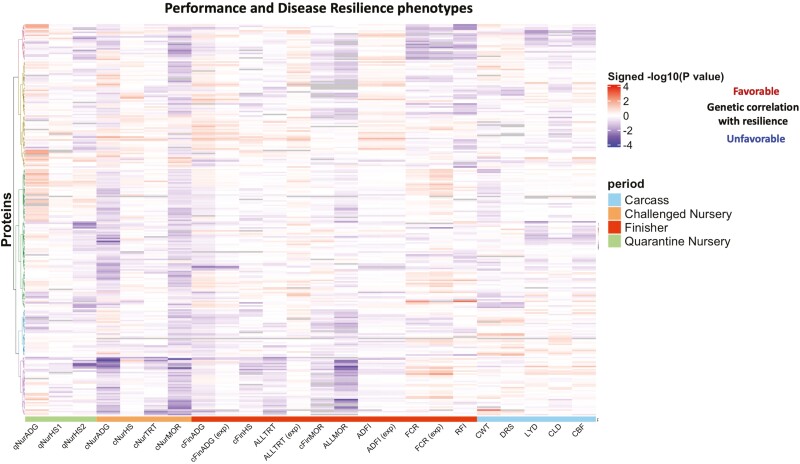
Heat map of the signed −log10(*P*-value) for estimates of the genetic correlation of protein abundance in blood of young healthy pigs with subsequent performance and disease resilience phenotypes for the survivor and expanded (exp) data sets. For trait abbreviations, see Table 1. The colors for the traits indicate the time period that the trait measured, where green represents quarantine nursery, orange the challenge nursery, red the finisher, and blue slaughter. Red/blue of the heat map value indicates that an increase in expression of that protein is favorable/unfavorably genetically correlated with the performance or disease resilience phenotype. Colors on the dendrogram identified different clusters based on ward.D clustering.

A heatmap of the signed −log10 of the *P*-values of estimates of genetic correlations for proteins with heritability estimates higher than 0.05 is shown in Figure 5. Since many estimates had large standard errors, the signed −log10(*P*-value) rather than the genetic correlation estimate was used to represent the strength of the genetic relationships. Plots of the signed −log10(*P*-value) against the estimate of the genetic correlation for each trait are in Supplementary Figure S3. Patterns of the signed −log10(*P*-value) were consistent for mortality in the different phases (Figure 5), as well as for feed conversion ratio (FCR) and residual feed intake (RFI). The patterns of genetic correlation estimates for the survivor and expanded data were highly similar, except for AllTRT.

### Gene set enrichment analysis of phenotypic associations

**Figure 6.**
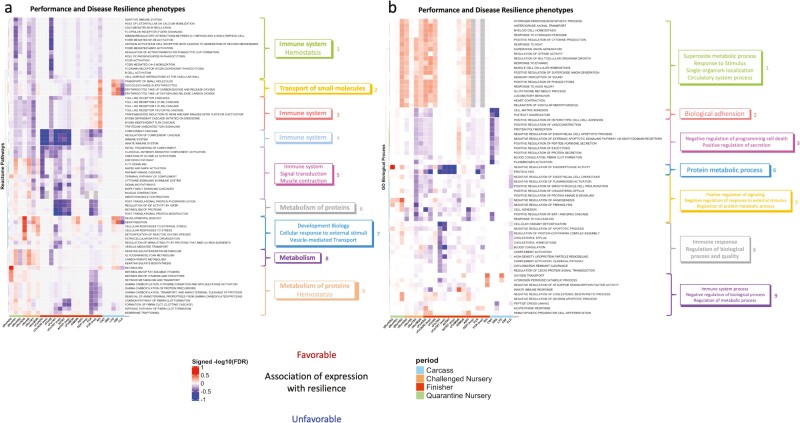
Heat map of the signed −log10 (FDR) for gene set enrichment analyses with proteins ranked based on the magnitude of the phenotypic association of their abundance with subsequent performance and disease resilience phenotypes for the survivor and expanded (exp) data sets. For trait abbreviations, see Table 1. Red/blue = an increase in expression of core enrichment proteins in this set was associated with better/poor performance. (a) REACTOME pathways (*n* = 72) that were significantly (FDR < 0.2) enriched among proteins ranked based on the magnitude of the association of their abundance with at least one phenotype trait. (b) GO Biological process (*n* = 63) that were significantly (FDR < 0.35) enriched among proteins ranked based on the magnitude of the association of their abundance with at least one phenotype trait. Colors on the dendrogram identified different clusters based on ward.D clustering.

To overcome the limitation of low statistical power to detect associations of the plasma proteome with performance and resilience traits for individual proteins, gene set enrichment analyses based on GO terms and ­REACTOME pathways were used to evaluate patterns in associations across proteins for each recorded phenotype. For these analyses, proteins were ranked based on the signed −log10(*P*-value) of their estimated association with the trait. The level of significance and direction of the enrichment of GO terms and REACTOME pathways that were significantly enriched for at least one trait are shown in Figure 6 for all recorded traits. For this purpose, liberal significance thresholds based on FDR were chosen to allow a meaningful number of terms or pathways to be included in the subsequent clustering analyses.

The 72 REACTOME pathways with FDR < 0.2 for enrichment of phenotypic associations for at least one trait separated into nine clear clusters, of which four (1-green, 3-red, 4-light blue, and 5-pink) were related to the immune system (Figure 6a). These clusters were unfavorably associated with disease resilience phenotypes, especially with health scores and the number of treatments. The REACTOME pathways in cluster 4 (light blue), which included the complement cascade process, had strong unfavorable associations with disease resilience in the finisher period, except with mortality. The REACTOME pathways in clusters 6 (grey), 8 (purple), and 9 (light brown) were primarily associated with metabolism and were favorably associated with phenotypes recorded in the quarantine and challenge nursery but unfavorably associated with phenotypes recorded in the finisher.

The 63 GO biological processes (BP) with FDR < 0.35 based on enrichment in phenotypic associations for at least one trait were separated into seven clusters (Figure 6b). The grey cluster contained several GO BPs related to immune response, such as complement activation, and was favorably associated with phenotypes recorded in the quarantine nursery but unfavorably associated with phenotypes recorded in the challenge nursery and finisher, especially with cFinTRT.

**Figure 7.**
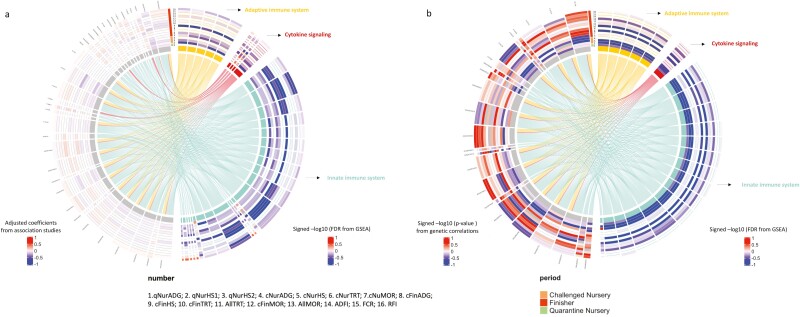
Chord diagrams of the relationships between proteins and enriched immune-related terms for the survivor data set. For trait abbreviations, see Table 1. The left half of the circle represents proteins and the right half immune-related terms that were enriched among proteins based on their phenotypic or genetic associations with performance and resilience phenotypes. Each layer of the concentric circles represents one numbered phenotype trait. (a) Chord diagram based on estimates of phenotypic associations of 35 proteins and 32 enriched REACTOME pathways (innate immune system: 23; adaptive immune system: 5; cytokine signaling: 4). (b) Chord diagram based on genetic correlation estimates of 23 proteins and 21 enriched REACTOME pathways (innate immune system: 15; adaptive immune system: 5; cytokine signaling: 1) is shown on the right side. Red/blue for the left halves indicates the scaled phenotypic regression coefficients (a) and genetic correlations (b) between protein abundance and phenotypic traits. Red/blue for the right halves indicates the signed −log10 (FDR) to enrich REACTOME pathways.

The 32 immune-related REACTOME pathways that were significantly (FDR < 0.2) enriched in phenotypic associations with disease resilience traits included 35 proteins, and their relationships and associations with all recorded phenotypes are illustrated as a chord diagram in Figure 7a. Among the significantly enriched immune-related pathways, pathways related to the innate immune system were found to be the most abundant (23), then pathways related to the adaptive immune system (5), and to cytokine signaling (4). All these proteins are involved in the innate immune system and most are also involved in the adaptive immune system and in cytokine signaling.

### Gene set enrichment analysis of genetic correlations

**Figure 8.**
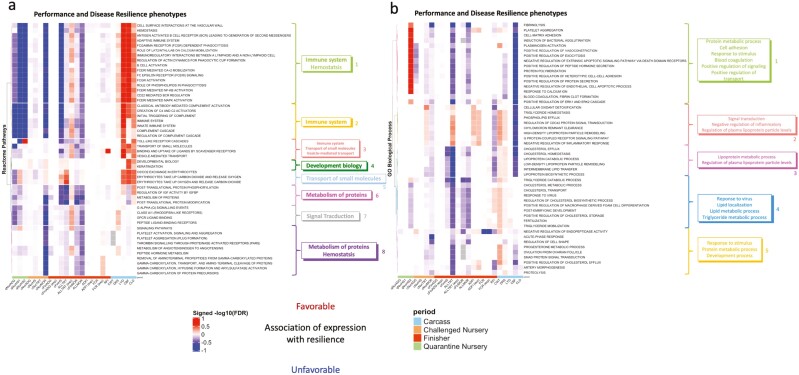
Heat map of the signed −log10 (FDR) for gene set enrichment analyses with proteins ranked based on magnitude of the phenotypic association of their abundance with subsequent performance and disease resilience phenotypes for the survivor and expanded (exp) data sets. For trait abbreviations, see Table 1. Red/blue = an increase in expression of core enrichment proteins in this set was genetically correlated with better/poor performance. (a) REACTOME pathways (*n* = 50) that were significantly (FDR < 0.2) enriched among proteins ranked based on the magnitude of the genetic correlation of their abundance with at least one phenotype trait. (b) GO Biological process (*n* = 49) that were significantly (FDR < 0.2) enriched among proteins ranked based on the magnitude of the genetic correlation of their abundance with at least one phenotype trait. Colors on the dendrogram identified different clusters based on ward.D clustering.

Figure 8 shows gene set enrichment results for estimates of genetic correlations for proteins with heritability estimates greater than 0.05. The proteins were ranked by the signed −log_10_(*P*-value) of their estimate of the genetic correlation with a trait. In total, 50 REACTOME pathways were significantly (FDR < 0.2) enriched, which separated into eight clusters (Figure 8a). The first three clusters (light green, yellow, and pink) were related to the immune system and had similar genetic relationships with the recorded phenotypes; they were unfavorably associated with disease resilience and performance traits, except with AllTRT (exp), but favorably associated with carcass traits. The REACTOME pathways in the green cluster were related to developmental biology and had low favorable genetic correlations with performance and resilience traits, except with CBF and CLC. The light blue cluster included pathways related to the transport of small molecules and showed favorable genetic correlations with carcass traits. The REACTOME pathways in the last three clusters included metabolism of proteins and signal transduction and showed unfavorable genetic correlations with disease resilience, except with AllTRT in the expanded data.

The 21 immune-related REACTOME pathways that were significantly (FDR < 0.2) enriched in genetic correlations with disease resilience traits included 15 innate immune system pathways, 5 adaptive immune system pathways, and 1 ­cytokine signaling pathway. In total, 23 proteins were involved in these pathways (Table 5), and their relationships and genetic correlations with all recorded phenotypes are illustrated as a chord diagram in Figure 7b. All proteins and immune-related pathways shown in Figure 7b overlapped with those identified based on phenotypic associations in Figure 7a. Based on genetic correlations, almost all significantly enriched immune-related pathways were unfavorably correlated with the recorded phenotypes, but the direction of estimates of genetic correlations of the abundance of the proteins involved in these immune-related pathways with the phenotypic traits was not consistent.

**Table 5.** List of proteins for immune system pathways for the chord plot (Figure 7b) based on genetic association studies.

Protein ID	Protein name	Gene name	No. of Immune system	No. of Innate immune system	No. of adaptive immune system	No.of cytokine signaling
A0A286ZKA5	C1q domain-containing protein	*C1QB*	6	6	0	0
A0A286ZSJ7	Complement C1q subcomponent subunit C (complement C1q subcomponent subunit C isoform 1)	*C1QC*	6	6	0	0
A0A286ZTC4	Ig-like domain-containing protein		20	14	5	1
A0A286ZVT2	Ig-like domain-containing protein		20	14	5	1
A0A286ZWN6	Ig-like domain-containing protein		20	14	5	1
A0A286ZXN4	Ig-like domain-containing protein		20	14	5	1
A0A287A6Q0	Vitamin K-dependent protein S	*PROS1*	3	3	0	0
A0A287A8V0	Ig-like domain-containing protein		20	14	5	1
A0A287AA42	Ig-like domain-containing protein		20	14	5	1
A0A287AG13	Apolipoprotein B-100	*APOB*	2	2	0	0
A0A287AKL0	Serum amyloid A protein		2	2	0	0
A0A287ASW4	Ig-like domain-containing protein		20	14	5	1
A0A287AXC8	Ig-like domain-containing protein		20	14	5	1
A0A287B3W7	Ig-like domain-containing protein		20	14	5	1
A0A287BFU6	Uncharacterized protein	*C1RL*	6	6	0	0
A0A287BH90	Complement component C9	*C9*	3	3	0	0
A7YX24	Gamma-synuclein	*SNCG*	6	6	0	0
F1RL06	Ig-like domain-containing protein	*LOC100523213*	20	14	5	1
F1RX35	Fibrinogen C-terminal domain-containing protein	*FGG*	2	2	0	0
F1S788	MACPF domain-containing protein	*C8A*	3	3	0	0
F1STC2	Ig-like domain-containing protein		20	14	5	1
F1STC5	Ig-like domain-containing protein		20	14	5	1
I3L728	Ig-like domain-containing protein		20	14	5	1

Figure 8b shows 49 GO BPs that were significantly enriched at FDR < 0.2 for at least one of the recorded phenotypes, which were separated into five clusters. None of the enriched GO BP related to immune response, but one was related to response to virus and response to the stimulus.

## Discussion

### Plasma proteome of young healthy pigs

The overall objective of this study was to explore the biological and genetic basis of the abundance of proteins in plasma from young, healthy pigs and investigate their associations with and potential as phenotypic and genetic indicators for disease resilience. Compared with previous studies on plasma proteins in pigs, this is the first study to explore the plasma proteome of young, healthy pigs on a large-scale. Previous studies focused on the genetic basis of specific proteins in the plasma of pigs. For example, Clapperton et al. (2009) studied the genetic basis of acute-phase proteins in the plasma of pigs raised in specific-pathogen-free and non-specific-pathogen-free environments, including alpha-acid glycoproteins, C-reactive protein, haptoglobin, and transthyretin. Ballester et al. (2020) investigated the genetic parameters of immunoglobulins and acute phase proteins (C-reactive protein and haptoglobin) in plasma from 8-week-old healthy Duroc piglets. The second novelty of this study is that this is the first to investigate phenotypic and genetic associations between the plasma proteome measured in a healthy condition with subsequent performance and resilience phenotypes under a disease challenge. The phenotypic and genetic associations were used to evaluate whether plasma protein levels measured in healthy piglets can be used as phenotypic or genetic indicators or predictors of disease resilience traits following concurrent exposure to multiple diseases, reflecting a severe disease challenge in a commercial environment.

### Genetic basis of the proteome in plasma from young healthy pigs

This study is the first to comprehensively investigate the genetic basis of the plasma proteome in young, healthy pigs by combining proteome abundance data with genome-wide SNP data. Abundances of nearly a quarter of the proteins (100) were found to be heritable (FDR < 0.10), with heritability estimates ranging from 0.17 to 1 (Supplemental Table 1). This reflects a strong impact of genetics on the plasma protein profile of young, healthy pigs. Litter effects were significant (FDR < 0.10) for only two proteins (L8B0W9 and L8AXL9, both are IgG heavy chains), explaining 18% and 21% of the phenotypic variance, respectively. The top four heritable proteins were the complement system proteins, with estimates of heritability close to 1, including complement factor H isoform a (A0A480TLF3, 71.7% missing), complement factor I isoform 1 preproprotein (A0A480P4D2, 63.0% missing), complement component C9 (A0A4X1T2W4, 46.7% missing), and complement C5a anaphylatoxin (A0A286ZKB4, 65.2% missing). The IgG heavy chain proteins were also highly heritable, with estimates up to 0.79.

In humans, using the mass spectrometry method, Johansson et al. (2013) found that the abundances of nearly one-fifth of the more than 1,000 peptides identified in plasma from around 1,000 individuals (at least 15 years of age) were significantly heritable (FDR < 0.05), with estimates ranging from 0.08 to 0.43. Liu et al. (2015) applied the SWATH mass spectrometry method to quantify the abundance of 342 proteins in plasma samples from 232 humans (between 38 and 78 years of age) and found that the abundance of 67 proteins had significant heritability (unadjusted *P*-value < 0.05), with estimates ranging from 0.21 to 0.66. In our study, estimates of heritability of the abundance of significantly heritable proteins in plasma from healthy piglets ranged from 0.17 to 1, i.e. higher than obtained in these previous studies in humans. The difference may be due to differences in the species, in age and uniformity of age when samples were collected, in health status, and the number of samples.

Interestingly, consistent with our finding of high heritability estimates of abundances of proteins of the complement system, Johansson et al. (2013) found the abundance of a peptide from the complement three protein in human plasma, coded by the *C3* gene, to have the highest heritability estimate (0.43). Also, in the study by Liu et al. (2015), abundance of complement factor H-related proteins one and three had among the highest heritability estimates, around 0.6, and abundances of other complement proteins, such as C3/C5 convertase (B9TSR8) and C4a anaphylatoxin (B0LFE9), also had higher estimates of heritability, around 0.55.

Consistent with our results, Liu et al. (2015) found that abundance of the IgG heavy chain protein had a high heritability (0.65) in human plasma. In a pig study, Ballester et al. (2020) estimated the heritability of abundance of IgG in plasma to be 0.65, while the estimate of the abundance of the acute phase protein haptoglobin was 0.40. The latter protein was also detected in our study, with an estimate of heritability of its abundance of 0.32. In another pig study, Clapperton et al. (2009) found the heritability of the abundance of haptoglobin in serum to be 0.23 in large white pigs raised in non-specific-pathogen-free environments. Our estimate of heritability for abundance of haptoglobin was between the estimates obtained in these two studies. In addition to sampling errors, differences may be due to differences in breeds used, age, health status, sample size, and our use of plasma rather than serum.

Monocyte differentiation antigen CD14 was another innate immune-related protein that was identified in our study. The CD14 protein acts as a co-receptor with the toll-like receptor TLR-4 and MD-2 to detect bacterial lipopolysaccharide (Kitchens, 2000; Tapping and Tobias, 2000). The estimate of heritability of abundance of the CD14 protein was 0.17 in our study, which was lower than the estimate of 0.33 in the blood of adult humans (Reiner et al., 2013).

Rao et al. (2015) found that the heritability of the abundance of apolipoprotein B-containing lipoproteins in blood of adult humans was 0.87, compared to our estimate of 0.44 for the heritability of the abundance of apolipoprotein B coded by the *APOB* gene. Although different types of apolipoproteins were detected and measured, estimates of heritability of the abundance of apolipoprotein were relatively high in both these studies. The main function of apolipoproteins is to transport lipids to cells in various tissues and their concentration in blood is an important indicator of cardiovascular diseases. Mice lacking apolipoprotein were shown to be more susceptible to bacterial infections (Peterson et al., 2008).

### Phenotypic associations of the proteome in plasma of young healthy pigs with performance and resilience phenotypes

In the phenotypic association studies between the abundance of specific proteins in plasma with concurrent and subsequent performance and resilience phenotypes, only a few proteins were identified to be significant after multiple test corrections (FDR < 0.35) because of limited statistical power (Table 3). To overcome this, gene set enrichment analyses were used, borrowing information across proteins involved in the same pathway or biological process. This identified many significantly enriched REACTOME pathways and GO biological processes and led to identification of more potentially relevant proteins (Figure 6), as discussed in the following. In Figure 6, the direction of the identified association of an enriched biological term with a phenotype was indicated to be favorable or unfavorable. A favorable direction of an association indicates that an increase in the abundance of core proteins associated with this term (not necessarily all proteins) was correlated with better performance or resilience. For the phenotypic association studies, immune-related REACTOME pathways (1-green, 3-red, 4-light blue, and 5-pink) and the metabolism of protein REACTOME pathway (6-grey) were unfavorably associated with performance and resilience phenotypes, especially for health scores and the number of health treatments. Immune-related GO biological processes (8-grey) were also unfavorably associated with phenotypes measured in the challenge nursery and finisher, especially with the number of treatments in the finisher. The direction of these results was opposite to expectations, as it is commonly understood that an increase in the abundance of immune-related proteins after exposure is associated with higher disease resilience. For example, Boehmer et al. (2011) found that the content of antimicrobial peptides and acute-phase proteins in the alveolar fluid of bovines increased after experimental induction of pneumonia with *Mannheimia haemolytica*. However, in our study, protein abundance was measured before exposure to disease, which may explain why a greater abundance of core proteins associated with immune-related REACTOME pathways and GO biological processes was associated with lower disease resilience after exposure to disease. A blood transcriptomics study of these same pigs by Lim et al. (2021) also suggested that piglets with greater expression of immune-related genes in whole blood before exposure tended to be less resilient after exposure to disease.

The immune system pathways that were associated with subsequent disease resilience clustered into three categories based on their biological function, as shown in Figure 7a, i.e. the innate immune system, the adaptive immune system, and cytokine signaling. The host’s innate and adaptive immune mechanisms work together to eliminate pathogens. Innate immunity represents general, nonspecific immunity, and is the first line of defence against non-autogenous pathogens through physical, chemical, and cellular mechanisms. Adaptive immunity, also called acquired immunity, occurs when the animal is exposed to a specific pathogen or is administered a vaccine, and can provide protection from that pathogen with subsequent exposure. Cytokines are a group of signaling proteins that are secreted by immune-related cells and can mediate and regulate the immune system. The proteins involved in these three immune system components are shown in Figure 7a, with details of the proteins provided in Table 6. The chord diagram displays the inter-relationships between proteins and pathways that were significantly enriched with phenotypes by gene set enrichment and shows that the number of significant pathways was largest for the innate immune system. This was as expected because protein levels were measured before the piglets were exposed to the disease challenge. However, several adaptive immune system pathways were significantly associated with subsequent disease resilience based on gene set enrichment, possibly reflecting exposure to minor pathogens or stress from weaning, transportation, mixing, and exposure to a new environment and diet, as also suggested by Lim et al. (2021) based on blood transcriptome analyses of these same pigs.

**Table 6:** List of proteins for immune system pathways for the chord plot (Figure 7a) based on the phenotypic association studies

Uniprot ID	Protein name	Gene name	No of immune system	No. of innate immune system	No. of adaptive immune system	No. of cytokine signaling
A0A286ZLR0	Ig-like domain-containing protein		20	14	5	1
A0A286ZSJ7	Complement C1q subcomponent subunit C (complement C1q subcomponent subunit C isoform 1)	*C1QC*	6	6	0	0
A0A286ZTC4	Ig-like domain-containing protein		20	14	5	1
A0A286ZVT2	Ig-like domain-containing protein		20	14	5	1
A0A286ZWN6	Immunoglobulin-like domain		20	14	5	1
A0A286ZXN4	Ig-like domain-containing protein		20	14	5	1
A0A287A6Q0	Vitamin K-dependent protein S	*PROS1*	3	3	0	0
A0A287A8V0	Ig-like domain-containing protein		20	14	5	1
A0A287AA42	Ig-like domain-containing protein		20	14	5	1
A0A287AG13	Apolipoprotein B-100	*APOB*	2	2	0	0
A0A287ASW4	Ig-like domain-containing protein		20	14	5	1
A0A287AXC8	Ig-like domain-containing protein		20	14	5	1
A2SW51	Monocyte differentiation antigen CD14 (myeloid cell-specific leucine-rich glycoprotein)	*CD14*	8	8	0	0
F1RL06	Ig-like domain-containing protein	*LOC100523213*	20	14	5	1
F1S788	MACPF domain-containing protein	*C8A*	4	4	0	0
F1SMJ1	Complement component C7	*C7*	4	4	0	0
F1SS24	Fibronectin	*FN1*	5	1	0	4
F1STC2	Ig-like domain-containing protein		20	14	5	1
F1STC5	Ig-like domain-containing protein		20	14	5	1
I3L728	Ig-like domain-containing protein		20	14	5	1
P82460	Thioredoxin (Trx)	*TXN*	1	1	0	0
A0A286ZKA5	C1q domain-containing protein	*C1QB*	6	6	0	0
A0A286ZMS2	Complement C1s subcomponent		6	6	0	0
A0A287BH90	Complement component C9	*C9*	4	4	0	0
A0A287A113	Spectrin beta chain	*SPTB*	3	0	0	3
A7YX24	Gamma-synuclein	*SNCG*	6	6	0	0
A0A287AFQ4	Lipocln_cytosolic_FA-bd_dom domain-containing protein	*C8G*	4	4	0	0
A0A287AQ20	Complement factor I isoform 1 preproprotein	*CFI*	3	3	0	0
F1RQW7	C3/C5 convertase (EC 3.4.21.43) (Complement C2, C2a fragment, C2b fragment)	*C2*	4	4	0	0
A0A287BFU6	Complement C1r subcomponent		6	6	0	0
F1RX35	Fibrinogen C-terminal domain-containing protein	*FGG*	2	2	0	0
F1S0J2	Uncharacterized protein	*C4BPA*	3	3	0	0
P26234	Vinculin (Metavinculin)	*VCL*	5	1	0	4
A0A287AKL0	Serum amyloid A protein		9	8	0	1
A0A287B3W7	Ig-like domain-containing protein		20	14	5	1

Based on the phenotypic association studies, the proteins involved in the significant immune-related pathways fell into two main classes: Ig-like domain-containing proteins and complement system proteins. Ig-like domain-containing proteins participate in all three components of the immune system (innate, adaptive, and cytokine signaling), while complement system proteins only participate in the innate immune system. Abundance of one of the Ig-like domain-containing proteins, F1STC5, as well as its related immune pathways, were found to be significantly unfavorably associated with a health score in the finisher, which suggests that piglets that had a greater abundance of the immune-related protein in plasma before exposure tended to be less resilient following exposure to disease. Transcriptome studies on these same pigs by Lim et al. (2021) reached the same conclusion. The complement system plays an important role in the innate immune system and builds a functional bridge between the innate and adaptive immune systems (Dunkelberger and Song, 2010). The complement system is a complex network of plasma and membrane-associated serum proteins. After being activated by pathogens, various complement components have bacteriolytic and cytolytic immune activity, lyse cells, mediate inflammation, regulate phagocytosis and immune response, and clear immune-related complexes (Sarma and Ward, 2011). In addition to Ig-like domain-containing proteins and complement system proteins, monocyte differentiation antigen CD14 and apolipoprotein B-100 were also among the proteins involved in significantly enriched immune-related pathways that were associated with disease resilience (Table 6). However, as individual proteins, these proteins were not significant in the phenotypic association analysis, illustrating the ability of gene set enrichment to identify additional proteins that are potentially relevant.

In addition to the proteins referred to above that were identified by gene set enrichment, several other proteins were found to be significantly associated with at least one trait after multiple test corrections (Table 3), most with ADG in the quarantine nursery. Among them, three proteins had moderate heritability (0.3–0.6), which were two uncharacterized proteins (A0A4X1U6L2 and A0A4X1U6T3) and Complement C4 gamma chain (A0A4X1VBD2). Complement C4 is a type of anaphylatoxin that plays an important role in immune response and host defence (Fritzinger et al., 1992). Anaphylatoxin can result in a local inflammatory response by triggering the release of substances by endothelial cells, phagocytes, or mast cells (Gennaro et al., 1986). Studies in humans have shown that complement C4 protein deficiency is related to systemic lupus erythematosus (Hauptmann et al., 1974) and type I diabetes mellitus (Dawkins et al., 1983; Mijovic et al., 1987). In a hepatic fibrosis study, Yang et al. (2011) found that complement component 4A in the serum of humans is a biomarker of hepatic fibrosis, with the grade of fibrosis increasing as the level of complement component 4A decreases. Complement C4 has not been studied much in pigs. Combined with results of the aforementioned studies on this protein in humans, the lower level of Complement C4 protein in plasma from pigs may indicate weak immune ability of pigs, which can negatively affect ADG when pigs are subjected to certain stressors such as infection, transport, mixing, and/or adaptation to solid feed post-weaning.

In general, it is impossible to determine whether the levels of proteins whose abundance during the quarantine nursery were associated with performance and disease resilience reflect base-line levels in young, healthy pigs, or are the result of response to environmental disturbances during or before quarantine nursery period. Gene set enrichment analyses of blood transcriptome data on these same pigs also identified both immune and stress response-related GO terms to be enriched among genes whose increased expression was unfavorably associated with both pre-and post-challenge traits (Lim et al., 2021). However, that study also identified GO terms related to protein localization and viral gene expression that were enriched among genes that were associated with reduced performance and health traits after but not before the challenge.

### Genetic associations of the proteome in plasma of young healthy pigs with performance and resilience phenotypes

This is the first study to estimate genetic correlations of the plasma proteome of young healthy pigs with their concurrent and subsequent performance and disease resilience phenotypes. Although for the latter, phenotypes on over 3,200 pigs were used through genomic relationships with the 912 pigs that had proteome data, only five plasma proteins were identified to have significant non-zero genetic correlations with at least one recorded phenotype after multiple test corrections, because of the high standard errors that are typically associated with estimates of genetic correlations (Table 4). None of these proteins overlapped with the proteins that were found to have significant phenotypic associations with these same traits (Table 3). Genetic correlations were not estimated for proteins with estimates of heritability less than 0.05 because standard errors of estimates of genetic correlations would even be larger for these proteins.

As for the phenotypic association studies, gene set enrichment analyses were also applied to the genetic correlation estimates, with proteins ranked based on the signed −log10(*P*-value) of the genetic correlation estimates with the recorded phenotypes. The pathways and GO biological processes that were significantly enriched based on genetic correlations were similar to those identified in the phenotypic association enrichment analyses. The three immune-related clusters (1-light green, 2-yellow, and 3-pink in Figure 8a) had similar patterns of genetic correlations between the enriched pathways and phenotype traits. The enriched immune-related pathways and metabolism of protein pathways were genetically unfavorably associated with performance and disease resilience traits, especially for traits measured during the quarantine and challenging nursery period, consistent with what we found based on phenotypic associations. However, in contrast to the phenotypic associations, the significantly enriched immune-related pathways were genetically favorably associated with carcass traits. The reason or relevance of this is not clear.

All proteins that had significant genetic correlations with one or more phenotypes had moderate to high heritability estimates (0.27–1, Table 4). Among them, the abundance of IgG heavy chain proteins had a strong negative genetic correlation (−0.68, L8B0V2) with ADG in the challenge nursery and a positive genetic correlation (0.85, L8AXM5) with mortality in the challenge nursery. Estimates of the litter effects for these two IgG heavy chain proteins were close to zero and not significant, which means the levels of IgG heavy chain proteins were not related to passive immunity. Further, abundance of complement component C9 was negatively genetically correlated with FCR in the expanded data (−0.24) and also had a very high heritability estimate (*h*^2^ = 1). C9 protein is one of the members of the complement system and plays an important role in innate immunity (Lint et al., 1980). When the complement system is activated, the C9 protein is polymerized and forms pores in the target cell membranes, causing cell lysis and death (Dudkina et al., 2016). A deficiency in C9 results in an inability to assemble the membrane attack complex, with a subsequent increase in susceptibility to infection (Zaoutis and Chiang, 2007). Combining the biological functions of the C9 protein and these previous studies, our result that an increase in the abundance of C9 protein (lower susceptibility to infection) in the plasma of young, healthy pigs is genetically associated with better feed efficiency under challenge is plausible.

Estimating the genetic correlation between protein abundance and phenotypes, as in our study, is considered the “gold standard” for identifying intermediate phenotypes, such as transcript or protein abundance, that is genetically correlated with a target phenotype (Gusev et al., 2016). Other studies have used so-called transcriptome-wide (Gusev et al., 2016) or proteome-wide association studies (Wingo et al., 2021) to identify such intermediate phenotypes. In TWAS or PWAS, a data set with the intermediate–omics phenotypes and whole-genome SNP genotypes are used to develop genomic predictions for each intermediate–omics phenotype, which are then used to predict the intermediate–omics phenotypes in a data set on individuals that have SNP genotypes and phenotypes for the target trait but no intermediate–omics phenotypes. Then, genetic associations between the intermediate–omics phenotypes and the target phenotype are identified based on the correlation between the genomic prediction for the intermediate–omics phenotype and the outcome phenotype. Although the resulting correlations are the result of genetics that affect both the intermediate and the target phenotype, they are not directly comparable to genetic correlations, as the latter quantify correlations between true genetic values for pairs of traits. In the present study, we used a large data set with SNP genotypes and target phenotypes, of which a subset also had proteomics data as the intermediate phenotypes. For such a data set, direct estimation of genetic correlations using maximum likelihood is well accepted to be the more powerful method to identify genetically correlated traits.

## Conclusions

This study combined quantitative analysis of population-level plasma proteome abundance data from blood collected on young, healthy pigs with phenotypes related to performance and disease resilience before and after their exposure to a polymicrobial natural disease challenge. Our results provide novel evidence that there is a genetic basis to differences in the plasma proteome of young, healthy pigs and that some of these differences are associated with performance and disease resilience following exposure, both phenotypically and genetically. Abundances in plasma prior to the disease challenge of proteins in pathways that are related to the immune system, especially the innate immune system, were unfavorably associated with performance and disease resilience after exposure to pathogens, both phenotypically and genetically. These results imply that pigs with unfavorable genetics for disease resilience either have higher base-line immune responses or produce a greater immune response to environmental disturbances that they are exposed to as young, visually healthy pigs. Regardless, our results show that the abundance of proteins in plasma from young, visually healthy pigs have potential as biomarkers for disease resilience and could be incorporated into breeding programs to improve selection for disease resilience.

## Supplementary Material

skad014_suppl_Supplementary_Figure_S1

skad014_suppl_Supplementary_Figure_S2

skad014_suppl_Supplementary_Figure_S3

skad014_suppl_Supplementary_Figure_S4

skad014_suppl_Supplementary_Table_S1

## Abbreviations

ADFI: average daily feed intake in the finisher
ADG: average daily gain
AllMOR: mortality (0 = pigs that survived; 1 = pigs that died) during challenge nursery and finisher periods
AllTRT: the number of individual parenteral antibiotic treatments in the challenge nursery and finisher, adjusted to 180 days since birth
BP: biological process
CBF: carcass back fat
cFin: finisher
cFinADG: average daily gain in the finisher
cFinHS: subjective health score at six weeks after entry into the finisher
cFinMOR: mortality (0 = pigs that survived; 1 = pigs that died) during the finisher period
cFinTRT: the number of individual parenteral antibiotic treatments in the finisher, adjusted to 100 days
CLD: carcass loin depth
cNur: challenge nursery
cNurADG: average daily gain in the challenge nursery
cNurHS: subjective health score at two weeks after entry into the challenged nursery
cNurMOR: mortality (0 = pigs that survived; 1 = pigs that died) during the challenge nursery period
cNurTRT: the number of individual parenteral antibiotic treatments in the challenge nursery, adjusted to 27 days
CWT: carcass weight
DRS: dressing percentage
EDTA: ethylenediaminetetraacetic acid
FCR: feed conversion ratio in the finisher
FDR: false discovery rate
GO: gene ontology
HS: health score
LYD: lean yield
NDCM: natural disease challenge model
PWAS: proteome wide association study
qNur: quarantine nursery
qNurADG: average daily gain in the quarantine nursery
qNurHS1: subjective health score at day 5 post entry into quarantine nursery
qNurHS2: subjective health score at the end of the quarantine nursery period
RFI: residual feed intake in the finisher
SD: standard deviation
SNP: single nucleotide polymorphism
TWAS: transcriptome wide association study
