# Supplementary figures and images for: Plasma protein levels of young healthy pigs as indicators of disease resilience
Source: J Anim Sci. 2023 Jan 13;101:skad014. doi: 10.1093/jas/skad014 (PMC9977353; doi:10.1093/jas/skad014)

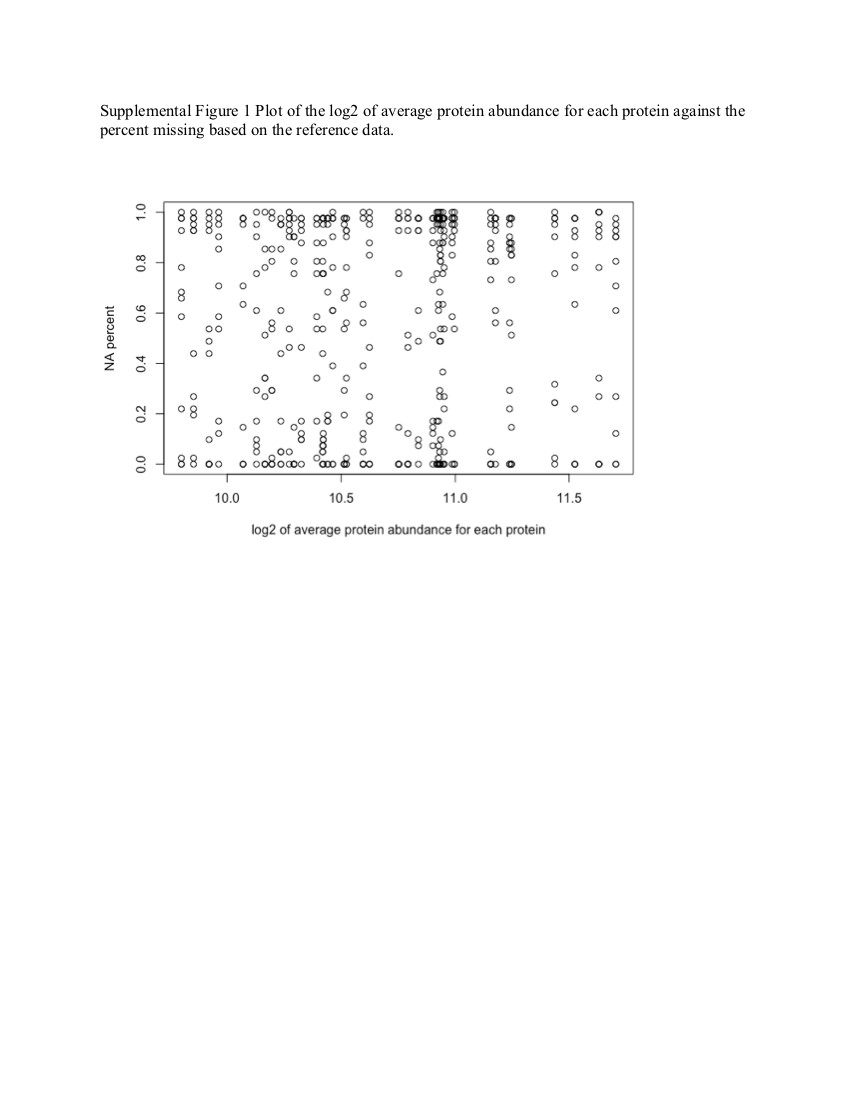

Supplement: skad014_suppl_Supplementary_Figure_S1 [file skad014_suppl_supplementary_figure_s1.jpeg]

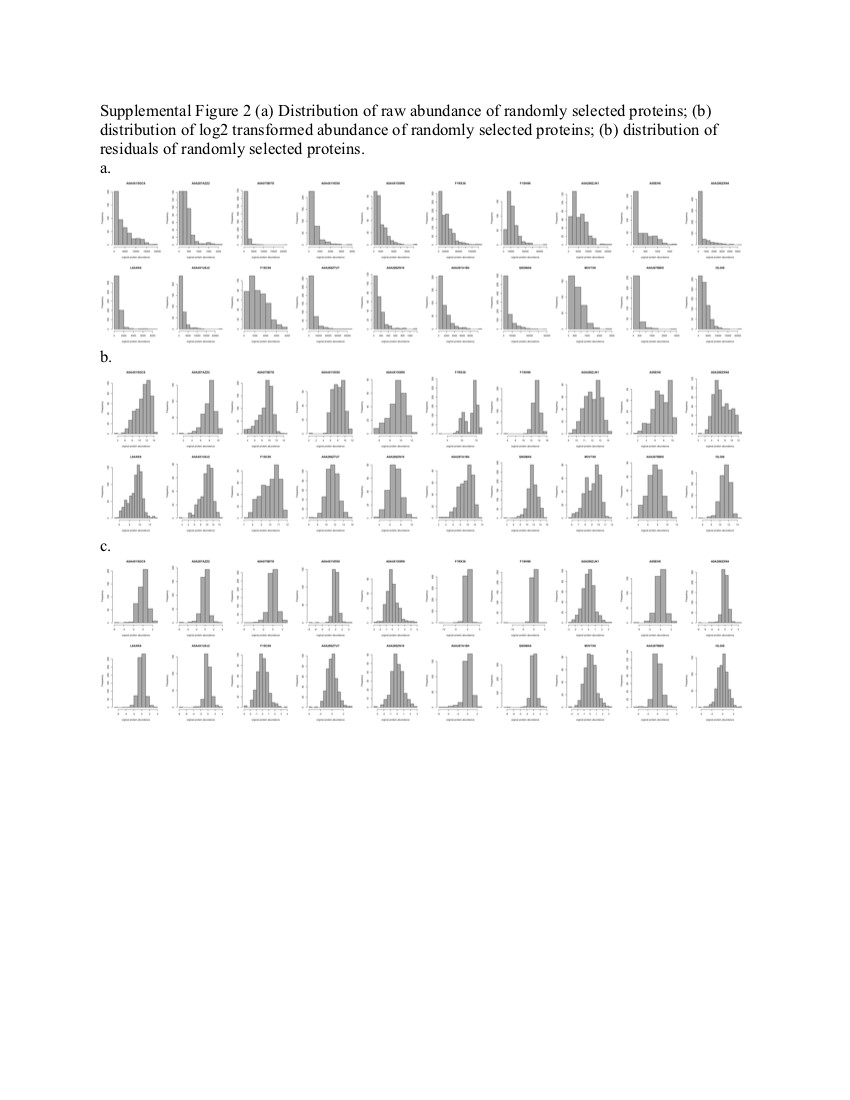

Supplement: skad014_suppl_Supplementary_Figure_S2 [file skad014_suppl_supplementary_figure_s2.jpeg]

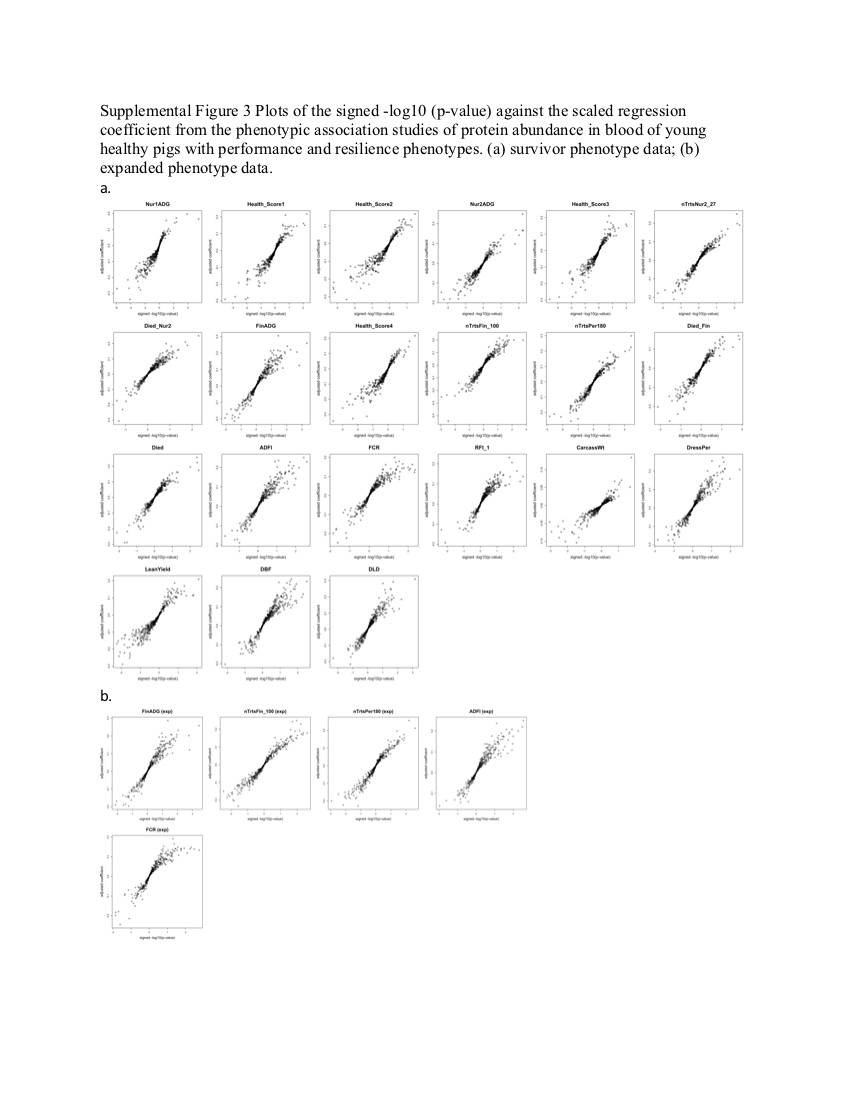

Supplement: skad014_suppl_Supplementary_Figure_S3 [file skad014_suppl_supplementary_figure_s3.jpeg]

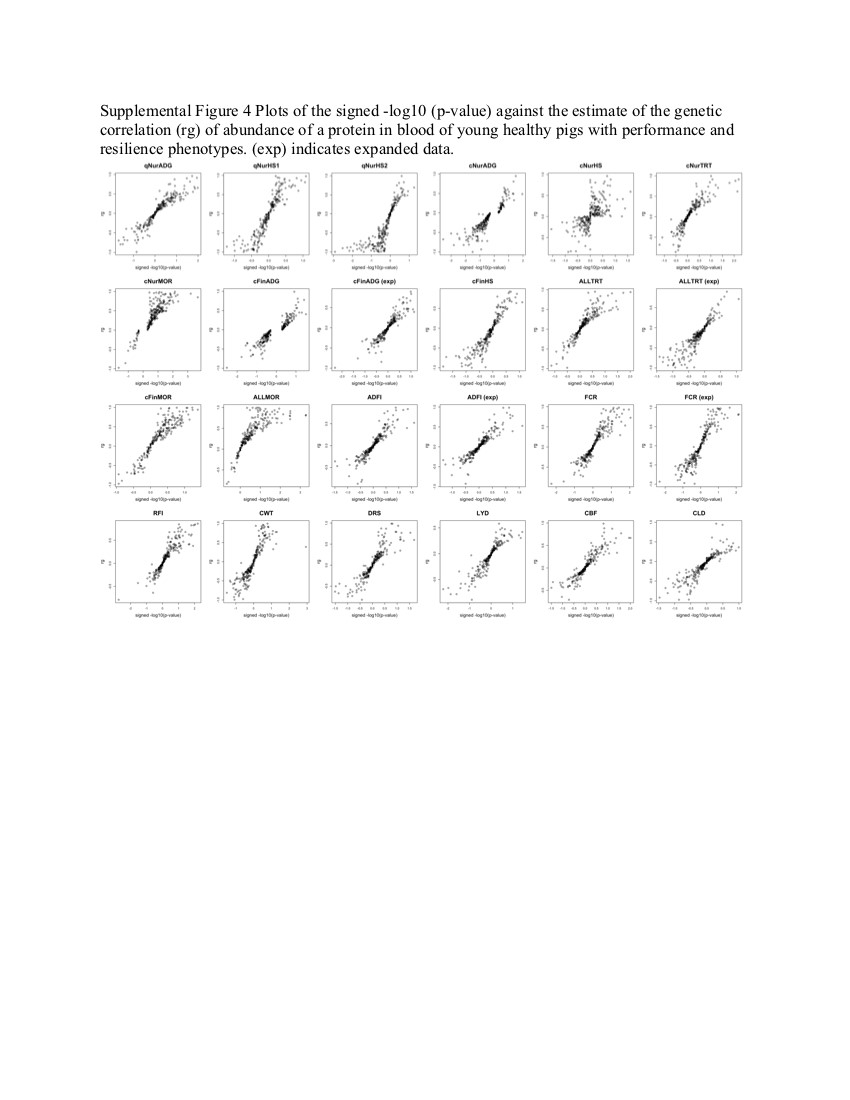

Supplement: skad014_suppl_Supplementary_Figure_S4 [file skad014_suppl_supplementary_figure_s4.jpeg]
